# Psychological Well-Being in Obstructive Sleep Apnea Syndrome Associated With Obesity: The Relationship With Personality, Cognitive Functioning, and Subjective and Objective Sleep Quality

**DOI:** 10.3389/fpsyg.2021.588767

**Published:** 2021-02-19

**Authors:** Federica Scarpina, Ilaria Bastoni, Simone Cappelli, Lorenzo Priano, Emanuela Giacomotti, Gianluca Castelnuovo, Enrico Molinari, Ilaria Maria Angela Tovaglieri, Mauro Cornacchia, Paolo Fanari, Alessandro Mauro

**Affiliations:** ^1^IRCCS, U.O. di Neurologia e Neuroriabilitazione, Ospedale S. Giuseppe, Istituto Auxologico Italiano, Piancavallo, Italy; ^2^“Rita Levi Montalcini” Department of Neuroscience, University of Turin, Turin, Italy; ^3^Laboratorio di Psicologia, IRCCS, Ospedale S. Giuseppe, Istituto Auxologico Italiano, Piancavallo, Italy; ^4^Department of Psychology, Università Cattolica del Sacro Cuore, Milan, Italy; ^5^IRCCS, U.O. di Riabilitazione Pneumologica, Ospedale S. Giuseppe, Istituto Auxologico Italiano, Piancavallo, Italy

**Keywords:** cognition, psychological well-being, temperament, sleep, obesity, OSA syndrome

## Abstract

Obstructive sleep apnea (OSA) syndrome severely affects psychological well-being. This syndrome frequently occurs in obesity; however, no previous study has investigated the level of psychological well-being in the case of OSA syndrome associated with obesity. In this work, we assessed the level of psychological well-being in fifty-two individuals affected by OSA syndrome and obesity through the Psychological General Well-Being Index. Moreover, we investigated the role of personality, cognitive functioning and attentional capabilities, subjective perception and objective measurement about sleeping, on the subjective perception of psychological well-being. Our sample reported a lower level of psychological well-being; the participants’ scores were below the normative cut-off in all components, except for depression symptoms. A lower expression of *harm avoidance* temperament and a lower level of daily sleepiness predicted a higher level of psychological well-being. Psychological well-being seemed to be severely affected in individuals affected by OSA syndrome and obesity. The temperament and subjective perception of daily alertness and sleepiness, rather than the syndrome severity, seemed to play a crucial role in the individual perception of the psychological well-being.

## Introduction

Obstructive Sleep Apnea (OSA) syndrome is a clinical condition characterized by periodic reductions (i.e., hypopnea) or cessations (i.e., apnea) of airflow due to narrowing of the upper airway during sleep. Also, frequent awakenings, episodes of oxyhemoglobin desaturation, and fluctuations of heart-rate, systemic and pulmonary artery pressure are observed. Affected individuals generally report to suffering from excessive daytime sleepiness, sleep fragmentation, and drowsiness, together with decreased energy, reduced level of concentration and alertness, and overall cognitive difficulties (i.e., Akashiba et al., 2002; Zhou et al., 2016; Simões et al., 2018; Angelelli et al., 2020). Crucially, these symptoms impact negatively on individuals’ efficacy in daily-life activities, such as working, driving, social interaction, and they increase the risk of (work- or road-) accidents (Vaessen et al., 2015). Consequently, in OSA syndrome, individuals generally experience a lower level of psychological well-being (Ohayon, 2003; Naismith et al., 2004; Saunamäki and Jehkonen, 2007; Harris et al., 2009; Sanchez et al., 2009; Iacono Isidoro et al., 2013; Hobzova et al., 2017), because of their difficulties about having a good and restorative sleeping (Hamilton et al., 2007; Weinberg et al., 2016) or because of the associated cognitive difficulties (Llewellyn et al., 2008; Wilson et al., 2013). Higher levels of anxiety and depressive symptoms (i.e., Ohayon, 2003; Naismith et al., 2004; Saunamäki and Jehkonen, 2007; Harris et al., 2009; Sanchez et al., 2009; Hobzova et al., 2017) are also registered. However, some of these symptoms (especially those related to a depressive mood) seem to be more related to the degree of daytime sleepiness, that is an effect of the disease, than to the degree of nocturnal hypoxemia, which expresses the severity of the disease (Naismith et al., 2004; Karkoulias et al., 2013). Moreover, Iacono Isidoro et al. (2013) highlighted that some individual factors might play a crucial role in the subjective perception of the psychological impact of OSA syndrome symptoms on psychological well-being, in line with evidence about beliefs and coping strategies (Dodge et al., 2012) as well as personality traits (Ryff, 2014).

OSA syndrome occurs frequently in obesity, as well as obesity is a high-risk factor for the development and progression of sleep apnea, even though the mechanisms linking obesity to the development and progression of OSA syndrome is still unclear (Schwartz et al., 2008). Obesity is a complex, multifactorial disease that develops from the interaction of genetic, metabolic, social, behavioral, and cultural factors. Thus, it might be expected that OSA syndrome in the context of obesity impacts severely individuals’ health (Veale et al., 2002; Iacono Isidoro et al., 2013). However, as in our knowledge, no study has investigated psychological well-being in individuals with both OSA syndrome and obesity. Indeed, studies focusing on OSA syndrome did not generally assess the participants’ level of obesity (i.e., Saunamäki and Jehkonen, 2007), as well as those studies in which psychological well-being (i.e., Carr and Friedman, 2005; Dierk et al., 2006; Heo et al., 2006; Simon et al., 2006; Manzoni et al., 2010; Jackson et al., 2015) was investigated in the case of obesity, did not generally assess sleeping quality or respiratory distress.

The present work aimed to describe the level of psychological well-being in a sample of individuals affected by OSA syndrome and obesity. To this aim, we used the Psychological General Well Being Index (Dupuy, 1984): it scores the psychological well-being as resulting from the components of anxiety, depressed mood, positive well-being, self-control, general health, and vitality. Moreover, because of the previous evidence about the impact of sleeping quality, cognition, and personality on the individual perception of psychological well-being (i.e., Ryff, 2014; Hamilton et al., 2007; Llewellyn et al., 2008; Dodge et al., 2012; Wilson et al., 2013; Weinberg et al., 2016), we explored the predictive role of these components in our sample’s experience. We assessed sleeping efficacy *quantitatively*, performing polysomnography, and *qualitatively*, through traditional self-report questionnaires. Neuropsychological tests focusing on the global functioning were administered to our participants to assess the cognitive efficiency; moreover, we investigated attentional abilities through a computerized task (Mueller and Piper, 2014; Scarpina et al., 2020). Finally, we profiled our participants’ personality referring to Cloninger et al. (1993)’s model, which postulates four temperamental traits: (i) *novelty seeking*, that defines the tendency to respond actively to novel stimuli leading to pursue rewards and escape from punishment; (ii) *harm avoidance*, corresponding to the tendency toward an inhibitory response to signals of aversive stimuli that lead to avoidance of punishment and non-reward; (iii) *reward dependence*, that is the tendency for a positive response to signals of reward to maintain or resist behavioral extinction; and, (iv) *persistence*, represented by the tendency to persevere in behavior, despite fatigue or frustration. The model also postulates three types of character, meaning self-concepts and individual differences in goals and values: (i) *self-directedness*, that is the ability to control, regulate and adapt behavior to fit the situation in accordance with individually chosen goals and values; (ii) *cooperativeness*, that accounts for individual differences in identification with and acceptance of other people; and, (iii) *self-transcendence*, which refers generally to the identification with everything conceived as essential and consequential part of a united whole. In our study, we used the Temperament and Character Inventory (Cloninger et al., 1993) to describe the level of expression for each temperament and character in our sample.

## Materials and Methods

### Participants

This study was approved by the Ethics Committee of the I.R.C.C.S. Istituto Auxologico Italiano (Reference number: 21C924_2019). All participants were volunteers who gave informed written consent before participating in the study; they were free to withdraw at any time.

Fifty-two individuals (32 females; 20 males; mean age in years = 54.61; *SD* = 9.52; range = 34–75; mean education in years = 11.88; *SD* = 3.82; range = 5–23) participated at this study. They were in-patients consecutively recruited at their admission to the institute, before an intensive hospital-based and medically managed rehabilitation program for weight reduction. Individuals were included in this study if at the admission: (i) they were affected by obesity, meaning a body mass index (BMI, defined as weight in kilograms divided by height in meters squared) over the value of 30; and (ii) they reported symptoms that might be referred to the OSA syndrome, such as: snoring loud enough to disturb their own or relatives’ sleep; waking up gasping or choking; intermittent pauses in breathing during sleep; excessive daytime drowsiness, with falls asleep during working or daily life activities. We excluded individuals who used C-PAP currently or in the past. Also, we excluded: smokers; individuals with a history of alcohol abuse; individuals with a history of gastrointestinal, cardiovascular, psychiatric, neurological disorders or any concurrent medical condition not related to obesity.

Overall, our sample of participants reported a mean BMI of 44.99 kg/m^2^ (*SD* = 9.33; range = 24.97–59.14).

### Materials

The Psychological General Well Being Index (PGWBI) (Dupuy, 1984; Grossi et al., 2006) was used to assess six dimensions (anxiety, depressed mood, positive well-being, self-control, general health, and vitality) of the psychological well-being. We profiled the temperament and the character of our sample through the Temperament and Character Inventory (TCI) (Cloninger et al., 1994; Martinotti et al., 2008). We assessed the participants’ global cognitive functioning through the Mini-Mental State Examination (Folstein et al., 1975; Magni et al., 1996), the Clock Drawing Test (Rouleau et al., 1992; Siciliano et al., 2016), and the Frontal Assessment Battery (Dubois et al., 2000; Appollonio et al., 2005). Also, the attentional resources and cognitive inhibition were measured through the Flanker’s Test from the PEBL Psychological Test Battery (Mueller and Piper, 2014; Scarpina et al., 2020).

Three questionnaires were used to rate the subjective perception of sleeping efficacy: the Epworth Sleepiness Scale (Johns, 1991; Manni et al., 1999) which focuses on the pathological daily sleepiness; the Pittsburgh Sleep Quality Index (Buysse et al., 2005; Curcio et al., 2013), which assesses sleep quality; and the Stanford Sleepiness Scale (Hoddes, 1972; Romigi et al., 2015), which quantifies the subjective level of alertness and sleepiness throughout the day. Finally, all participants underwent a full-night polysomnography, which is routinely performed in our institute (i.e., Zibetti et al., 2017). The Apnoea–Hypopnoea Index (AHI), computed according to the number of apnea and hypopnea events per hour of sleep, was used as index of sleep apnea severity. Following the standard criteria (Ruehland et al., 2009), a value below of the cut-off of 5 was considered in the range of normality; values between the range 5 ≤ AHI < 15 defined a mild sleep apnea; the range 15 ≤ AHI < 30, a moderate apnea; values of AHI ≥ 30 defined a severe apnea. Details about all the measurements are reported in the Supplementary Material.

### Data Analysis

Preliminary, psychological questionnaires and neuropsychological tests were scored accordingly to the seminal works and compared to the normative data. About the computerized test of PEBL Flanker’s Test (Mueller and Piper, 2014), we computed behavioral indexes suggesting the presence of attentional difficulties (Scarpina et al., 2020). For details, consult the Supplementary Material.

To describe the level of the psychological well-being in our sample, an independent sample *t*-test was performed for each subscale of the PGWBI in order to compare the scores reported by our sample and the normative score for the Italian population reported in Grossi et al. (2006).

Successively, we performed a linear regression analyses to verify the role of temperament, cognitive functioning, and sleep characteristics on the level of psychological well-being in our sample. Thus, we preliminary analyzed the correlation and directionality of the data to formulate the statistical model: the Spearman’s correlation coefficient was computed between the PGWBI total score and those factors which would be included in the statistical model, that were: (i) the demographical characteristics of *Age* and *Education*, as well as the *BMI*; (ii) the scores relative to the four temperamental traits from the Temperament and Character Inventory; (iii) the score at the neuropsychological tests (Mini Mental State Examination, Clock Drawing Test, Frontal Assessment Battery, and Flanker’s Test - RT index and Accuracy index); (iv) the polysomnographic components; and (v) the scores at the questionnaires of Epworth Sleepiness Scale, Pittsburgh Sleep Quality Index and Stanford Sleepiness Scale. A nonparametric measure (i.e., the Spearman’s correlation) was used since most of the factors were not normally distributed, as usually observed in the case of questionnaires’ scores. Those factors significantly associated with the PGWBI total score (*p* ≤ 0.05) were further investigated with the linear regression model, about which we reported the R-squared as a goodness-of-fit measure; also, we evaluated the significance of the model through *F*-value and the *p*-value. Finally, we verified the relative contribution of the factors included in the statistical model with the independent variable (i.e., the PGWBI total score). About each of them, we reported the variance inflation factor (VIF) as measure of multicollinearity.

Further analyses focused on the OSA syndrome severity (Supplementary Table 1) and the obesity severity (Supplementary Table 2), as well as those analyses investigating possible differences in the individual factors between the different levels of OSA syndrome severity (Ruehland et al., 2009; Supplementary Table 3) are reported in the Supplementary Material.

## Results

In Table 1, mean, standard deviation, and range for all the measurements were reported.

**TABLE 1:** Minimum (Min) and maximum (Max) score, mean, standard deviation (*SD*), and values range are reported for all the measurements.

		Min-Max score	Mean	*SD*	Range
**Psychological well being:** Psychological general well being index					
Anxiety		0–25	14.17	5.19	1–24
Depression		0–15	11.25	3.12	2–15
Positive well-being		0–20	9.09	4.06	1–17
Self-control		0–15	10	3.27	3–15
General healthy		0–15	7.86	3.11	2–14
Vitality		0–20	8.84	4.31	1–17
Total score		0–110	61.23	19.8	19–100
**Personality:** Temperament and character inventory					
Novelty seeking		0–175	101.8	12.6	81–137
Harm avoidance		0–165	97.65	15.9	72–131
Reward dependence		0–150	101.51	11.05	75–125
Persistence		0–175	120.09	17.08	73–154
Self-directedness		0–200	134.63	19.76	88–180
Cooperativeness		0–180	135.11	14.66	108–189
Self-transcendence		0–130	73.75	13.92	42–100
**Cognitive functioning**					
Mini mental state examination		0–30	28.54	1.31	25–30
Clock drawing test		0–10	8.9	1.98	0–10
Frontal assessment battery		0–18	16.68	1.4	12–18
Flanker’s test	RTs	–	1.066	0.033	0.99–1.14
	Accuracy	–	1.01	0.039	0.92–1.12
**Sleep - quantitative measurement: polysomnography**					
Number of apnea/hypopnea events per hour of sleep (Apnea/Hypopnea Index – AHI)		–	47.57	34.62	3.1–150.2
Number of hypopnea events per hour of sleep (Hypopnea Index – HI)		–	35.21	31.78	0.3–108.2
Number of apnea events per hour of sleep (Apnea Index – AI)		–	24.88	21.55	0.4–87.8
Number of blood oxygen desaturation events per hour of sleep		–	45.25	32.58	3.8–136.5
Time with SaO_2_ <90% (% of total sleep time)		–	60.26	77.47	0.9–554
Average minimum SaO_2_ during desaturations (%)		–	83.58	4.29	71–90
Minimum oxygen saturation (%)		–	68.42	11.29	46–87.4
**Sleep - subjective evaluation**					
Stanford Sleepiness Scale		0–7	2.75	1.01	1.11–5.33
Pittsburgh Sleep Quality Index		0–21	7.57	3.1	1–13
Epworth Sleepiness Scale		0–24	8.57	5.4	0–23

**FIGURE 1:**
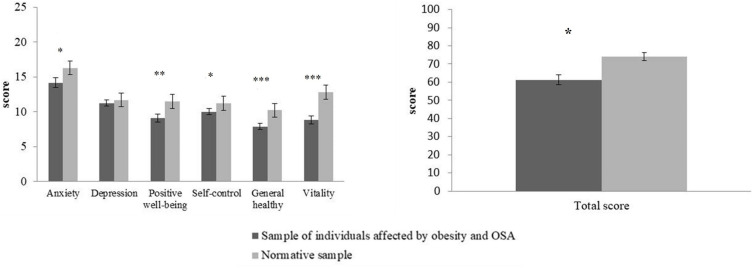
For each subscale (right panel) and the total score (left panel) relative to the Psychological General Well Being Index (Dupuy, 1984), mean (*y* axis) and standard deviation (vertical lines) are shown about the sample of individuals affected by OSA syndrome and obesity (dark gray bars) and the normative sample (light gray bars; data from Grossi et al., 2006).* indicates a *p*-value < 0.05; **p-value = 0.001; ***p-value < 0.001.

Focusing on the PGWBI, in all subscales, except for the level of depression, our sample reported lower scores in comparison with the normative sample (*N* = 76; age range = 50–54) (Grossi et al., 2006), as show in Figure 1. Thus, as suggested by the global score, our sample described a lower level of psychological well-being.

No significant relationship emerged between the *PGWBI global score* and the demographical characteristics of *Age* [ρ(52) = 0.05; *p* = 0.68] and *Education* [ρ(52) = 0.06; *p* = 0.66], or with the level of *BMI* [ρ(52) = −0.04; *p* = 0.75].

In Table 2, the results relative to the relationship between the *PGWBI total score* and the scores relative to the temperament, cognitive functioning, subjective perception and objective measurement of sleeping were reported.

**TABLE 2:** Results relative to the relationship between the PGWBI total score and the scores relative to the personality (Temperament and Character Inventory), the cognitive functioning (the neuropsychological assessment), the objective assessment of sleeping (polysomnography) and the subjective sleeping functionality (sleep – subjective evaluation: questionnaires).

Personality: Temperament and character inventory					
		**Novelty seeking**	**Harm avoidance**	**Reward dependence**	**Persistence**

PGWBI Total Score	ρ(p)	0.17 (0.22)	−0.47 (**<0.001)**	−0.11 (0.41)	0.1 (0.44)

**Cognitive functioning: Neuropsychological assessment**						

		**Mini mental state examination**	**Clock drawing test**	**Frontal assessment battery**	**Flanker ‘s test**	
					**RT**	**Accuracy**

PGWBI Total Score	ρ (p)	0.15 (0.26)	0.12 (0.38)	0.24 (0.08)	0.076 (0.59)	−0.02 (0.84)

**Sleep - quantitative measurement: Polysomnography**								

		**AHI**	**HI**	**AI**	**Blood O2 desaturation events**	**SpO2 < 90%**	**Mean minimum O2 saturation**	**Minimum O2 saturation**

PGWBI Total Score	ρ (p)	0.02 (0.88)	0.15 (0.29)	0.12 (0.42)	0.13 (0.34)	0.26 (0.06)	−0.16 (0.26)	−0.08 (0.57)

**Sleep - subjective evaluation**				

		**Epworth Sleepiness Scale**	**Pittsburgh Sleep Quality Index**	**Stanford Sleepiness Scale**

PGWBI Total Score	ρ (p)	−0.36 (**0.008)**	−0.37 (**0.006)**	0.58 (**<0.001)**

**n* = 52; In bold, significant results.*

We observed a significant negative relationship only with the score relative to the *harm avoidance* temperament, while no significant relationship emerged with the scores relative to the other temperamental traits (*p* ≥ 0.22). Thus, those participants affected by OSA syndrome and obesity with a lower expression of harm avoidance temperament reported a higher level of psychological well-being. No significant relationship between the *PGWBI total score* and the scores relative to the neuropsychological tests emerged (*p* ≥ 0.08). However, the role of a possible ceiling effect, as suggested by the data reported in Table 1, should be considered. No significant relationship emerged with any of the neurophysiological parameters from the polysomnography (*p* ≥ 0.06). Instead, all the subjective measures about the sleep efficacy were significantly related to the *PGWBI total score* (*p* ≤ 0.008). Thus, in the linear regression model we included the scores relative to the subjective sleeping questionnaires (*Epworth Sleepiness Scale*, *Pittsburgh Sleep Quality Index*, and *Stanford Sleepiness Scale*) and the score relative to the *harm avoidance* temperament, as predictors of the *PGWBI total score*. According to the results reported in Table 3, a higher global score at PGWBI, suggesting a higher level of psychological well-being, was significantly predicted by a lower score relative to the harm avoidance temperament, and a lower perception of daily sleepiness measured through the Stanford Sleepiness Scale. The other factors included in the model were not statistically significant.

**TABLE 3:** Results relative to linear regression model for the PGWBI total score.

Predictor	*B*	*t*	*p*	Variance inflation factor
Epworth Sleepiness Scale	−0.09	−0.19	0.84	1.42
Pittsburgh Sleep Quality Index	−0.58	−0.72	0.47	1.32
Stanford Sleepiness Scale	−8.94	−3.23	**0.002**	1.66
Harm avoidance temperament (from the Temperament and Character Inventory)	−0.31	−2.08	**0.043**	1.21

*R square = 0.43; *F*(4,51) = 8.96; *p* < 0.001. In bold, significant results.*

## Discussion

We aimed to describe the psychological well-being of individuals affected by OSA syndrome and obesity, and to assess the role of temperament, cognitive functioning, and subjective and objective sleeping efficacy on its perception.

We observed a lower level of psychological well-being, measured through the Psychological General Well Being Index (Dupuy, 1984), when our sample’s scores were compared with the Italian normative data (Grossi et al., 2006). This result seemed in agreement with Iacono Isidoro et al. (2013)’s study, in which the same questionnaire was used in the case of Italian individuals affected by OSA syndrome. The authors described their sample as suffering from moderate distress, since they reported a total score mean of 70.9 (*SD* = 16), ranging from 20 to 101, at the Psychological General Well Being Index (Dupuy, 1984). This score seemed to be higher in comparison with our sample’ score. However, it should be considered the role of obesity. Indeed, a level of BMI higher than 30 was an inclusion criterion in our study; according to the BMI range, individuals from severe obesity – Class II to very severe obesity – Class III (WHO, 1995) were included in our sample. Instead, Iacono Isidoro et al. (2013) did not specify the BMI as an inclusion/exclusion criterion in their study. As results, their sample’s BMI ranged from 17.3 to 57.8. Thus, not only individuals affected by different levels of obesity, but also individuals with healthy-weight as well as underweight individuals might have been included in the sample. Interestingly, we did not report any significant relationship between the level of psychological well-being and the level of obesity, as registered in other studies (i.e., Dierk et al., 2006; Manzoni et al., 2010; Iacono Isidoro et al., 2013), but in disagreement with other ones, in which psychological difficulties were reported to be higher for higher levels of obesity (i.e., Carr and Friedman, 2005; Heo et al., 2006; Simon et al., 2006; Jackson et al., 2015). Nevertheless, some cautions should be taken in confronting different studies, because the role played by social and cultural factors, which may, in turn, affect the perception of psychological well-being (Dierk et al., 2006). On the other hand, an ongoing debate is still in the literature about the meaningful and useful of the individual’s BMI as an index of level of obesity severity in the context of psychological and social studies (Schwartz and Brownell, 2004; Vieira et al., 2012). Our sample reported a lower score in almost all components of the Psychological General Well Being Index (Dupuy, 1984), except for the number of depressive symptoms, about which we observed a score in line with the normative sample (Grossi et al., 2006). This result seemed in agreement with previous evidence, such as Pillar and Lavie (1998), but in disagreement with other ones (i.e., Harris et al., 2009) about OSA syndrome and depression. Nevertheless, it should be considered that some of the symptoms typically observed in this clinical condition, such as physical fatigue, cognitive slowness and memory loss, headaches, restless sleep, mood changes, decreased sexual interest, may resemble symptoms associated with the depressive condition, challenging clinicians in diagnosing the presence of the psychiatric disease (Harris et al., 2009).

In our study, we also registered that a lower level of daily sleepiness and a lower expression of harm avoidance temperament predicted a higher level of psychological well-being in individuals affected by OSA syndrome and obesity. About the first component, our results, which relied on the score reported by participants at the Stanford Sleepiness Scale (Hoddes, 1972), were globally in line with the Iacono Isidoro et al. (2013): the authors observed that higher scores registered at the Epworth Sleepiness Scale (Johns, 1991), were related to the lower level of psychological well-being, as registered in our analyses. However, the authors did not report statistical results about the predictive role of the score at the Epworth Sleepiness Scale on the PGWBI score. Moreover, Iacono Isidoro et al. (2013) reported that the AHI index from the polysomnography, which represents an objective measurement about sleep quality and efficacy, did not predict the subjective perception of the psychological well-being, in line with our results. Also, our results and those provided by Iacono Isidoro et al. (2013) were in agreement in reporting no difference in the psychological well-being between different levels of sleep apnea severity (Ruehland et al., 2009). Finally, we also observed no significant relationship between the scores relative to the three questionnaires - measuring the subjective perception of daily sleeping and sleeping efficacy – and the Apnea/Hypopnea Index registered in the polysomnography (see Supplementary Material). Overall, all these results seem to confirm that lower psychological well-being might be not strictly related to the OSA severity. Instead, how individuals perceive the impact of syndrome on their sleeping and daily functioning may play a significantly crucial role. Thus, in this vein, objective measurements about sleeping quality, as reported in the present study as well as in Iacono Isidoro et al. (2013), might be mandatory not only when individuals spontaneously recognize and report to suffer from daily sleeping and decreased alertness, suggesting possibly OSA syndrome, but also in all these conditions in which such difficulties might emerge due to other clinical conditions, such as obesity.

The second component that predicted higher levels of psychological well-being was a lower level of *harm avoidance*. In other words, those affected individuals showing a carefree, courageous, and optimistic way to approach their health-related condition, might experience a higher level of psychological well-being. Within the sample, we observed that the majority of our participants reported a higher score in the scale relative to the *persistence* trait, although the other three temperamental traits seemed to be under-expressed. It was reported that a high level of *persistence* may play a protective effect on the emotional functioning, reducing negative emotions and increasing positive ones, especially in the case of lower expressions of *harm avoidance* (Cloninger et al., 2012). We assessed our participants before to start an intensive hospital-based and medically managed rehabilitation program for weight reduction. This medical choice may be embraced especially by those individuals who are highly persistent despite the perceived distress and more prone to pro-health activities. Also, it may depend on external solicitations, such as general practitioners and/or relatives, suggesting a rewards-dependent temperament. Nevertheless, about the high expression of persistence, “*its value depends on a complex set of adaptive processes, both internal and external, and how well these forces that push and pull on the person are integrated and balanced. Being highly persistent is likely to lead to intense mixtures of positive and negative emotions, which can lead to high achievement, much anxiety, or both*” (Cloninger et al., 2012; p. 8). Indeed, our participants reported higher levels of anxious (and not of depressive) symptoms: this result, which was in line with previous evidence (Saunamäki and Jehkonen, 2007; Gupta and Simpson, 2015), might be related to the higher expression of the persistence as temperamental trait. As in our knowledge, two previous studies (Sforza et al., 2002; Fìdan et al., 2013) described the personality according to Cloninger et al. (1993)’s model in individuals affected by OSA syndrome. Other studies described personality in individuals affected by this syndrome referring to other psychological models, such as the Eysenck and Eysenck (1975)’ model in So et al. (2015), and the behavioral inhibition system/behavioral activation system (BIS/BAS) model (Carver and White, 1994) in Moran et al. (2011). The two studies in which Cloninger et al. (1993)’s model was used reported heterogeneous results: Sforza et al. (2002) observed that individuals with OSA syndrome mostly showed a higher expression of novelty-seeking behavior in comparison with healthy adults, but no difference emerged in comparison with snoring individuals; instead, Fìdan et al. (2013) registered no difference in the four temperamental traits between individuals affected by OSA syndrome and healthy individuals. However, focusing on the obesity, we might observe that Sforza et al. (2002) assessed individuals with a level of BMI of 30.4 (*SD* = 0.7); similarly, Fìdan et al. (2013) reported a level of BMI of 30.9 (*SD* = 4.9) about their sample. Thus, in both these studies, individuals with low-risk obesity were assessed. Instead, we enrolled individuals with high-risk obesity.

In our work, we performed a global assessment of cognitive functioning. Our sample did not show pervasive cognitive difficulties or dementia. However, as reported in the Supplementary Material, we verified the presence of attentional difficulties, in accordance with previous evidence (Akashiba et al., 2002; Vaessen et al., 2015; Zhou et al., 2016; Simões et al., 2018; Angelelli et al., 2020) about OSA syndrome. It is worthy to note that overall cognitive difficulties were not assessed extensively in the literature. However, we would emphasize the importance to adopt neuropsychological tests to measure cognitive functioning, since a mismatch between the subjective perception (i.e., an efficient attentional functioning in daily-life) and the objective assessment (i.e., lower attentional capabilities measured through neuropsychological tests) might be observed.

In this article, we described the level of psychological well-being in the case of OSA syndrome and obesity, which was found to be severely decreased in the assessed sample. Specifically, we reported that the temperament and the subjective perception of daily alertness and sleepiness, rather than the syndrome severity, seemed to play a crucial role in the individual perception of the psychological well-being. However, since we performed an observational cross-sectional study, we cannot furnish any conclusive information about the relationship between OSA syndrome and obesity, as well as their specific role on the individuals’ psychological well-being perception. Indeed, to this aim, future studies in which different levels of obesity will be compared with healthy-weight condition with and without OSA syndrome should be performed. Moreover, it should be considered that in our study, because of the clinical procedure described in the Supplementary Material, participants were enrolled based on the suspicion of OSA syndrome according to the subjective symptoms, rather than its objective diagnosis, which grounds on the AHI registered at the polysomnography, Our findings might extend our understanding about how personality, subjective and objective measures of sleep quality, and cognitive functioning interact with the psychological well-being of affected individuals.

## Data Availability Statement

The datasets presented in this article are not readily available because the data are restricted according to the Ethical Commission’s indications. Requests to access the datasets should be directed to corresponding author (FS).

## Ethics Statement

The studies involving human participants were reviewed and approved by the IRCCS Istituto Auxologico Italiano. The patients/participants provided their written informed consent to participate in this study.

## Author Contributions

FS and AM conceived the study. FS supervised the entire study, performed the statistical analyses, and wrote the manuscript. IB and SC performed the data collection. LP, EG, IMAT, and MC selected the participants and performed the clinical assessment. GC and EM supervised the psychological assessment. PF and AM supervised the clinical assessment. All authors revised the manuscript.

## Conflict of Interest

The authors declare that the research was conducted in the absence of any commercial or financial relationships that could be construed as a potential conflict of interest.

## Supplementary Material

Supplementary Figure 1 About the Stanford Sleepiness Scale, we report mean and standard deviation for each time hour; vertical lines represent standard deviation. a.m., ante meridiem; p.m., post meridiem.

Supplementary Figure 2 About the Flanker’s Test, we report mean and standard error (vertical lines) for each experimental condition (*x*-axis) for the Reaction Time (RT) expressed in milliseconds (left panel) and the Accuracy in percentage (right panel). * indicates a significant difference (*p* < 0.05), according to the *post hoc* analyses.

Supplementary Table 1 The relationship between the AHI index and BMI, the scores relative to the personality (i.e., Temperament and Character Inventory), cognitive functioning (i.e., neuropsychological tests), and the subjective characteristics (self-report questionnaires) about sleeping; *n* = 52.

Supplementary Table 2 The relationship between the BMI and the scores relative to the personality (i.e., Temperament and Character Inventory), cognitive functioning (i.e., neuropsychological tests), and the subjective characteristics (self-report questionnaires) about sleeping. We report again the relationship with the AHI index, as shown in Supplementary Table 1. *n* = 52. In bold, significant relationships.

Supplementary Table 3 Mean and standard deviation (*SD*) for each assessed factors are reported for the different levels of sleep apnea, along with the statistical results.
