# Supplementary material for: Psychological Well-Being in Obstructive Sleep Apnea Syndrome Associated With Obesity: The Relationship With Personality, Cognitive Functioning, and Subjective and Objective Sleep Quality
Source: Front Psychol. 2021 Feb 19;12:588767. doi: 10.3389/fpsyg.2021.588767 (PMC7933550; doi:10.3389/fpsyg.2021.588767)
Supplement: Supplementary Figure 1 — About the Stanford Sleepiness Scale, we report mean and standard deviation for each time hour; vertical lines represent standard deviation. a.m., ante meridiem; p.m., post meridiem. [file Data_Sheet_1.docx]

**--- Supplementary Materials –**

**METHODS - OUTCOME: DETAILS**

**Psychological well-being.** The Italian version (Grossi et al., 2006) of the Psychological General Well Being Index (PGWBI) (Dupuy, 1984), that is routinely adopted in our Institute at the all in-patients’ admission, was used in this study to assess the psychological well-being. This questionnaire produces a self-representation of intra-personal affective or emotional states mirroring a sense of subjective well-being or distress; in other words, a subjective perception of well-being. The questionnaire explores six domains through 22 items: anxiety, depressed mood, positive well-being, self-control, general health and vitality. Each domain is defined by a minimum of three or a maximum of five items. The scores for all domains is summarized to provide a summary score, which reaches a maximum of 110 points. The higher is the score, the higher the level of psychological well-being.

**Temperamental traits.** The Italian version (Martinotti et al., 2008) of the Temperament and Character Inventory (TCI) (Cloninger et al., 1994)^0^ was used to picture the temperamental profile of our sample. This self-questionnaire consists of 226 items. According to the Cloninger and colleagues (1993), the questionnaire measures four traits: novelty seeking; harm avoidance; reward dependence; and persistence. Moreover, it measures three characters: self-directedness, cooperativeness, and self-transcendence.

**Cognitive functioning.** All these tests were assessed by an expert neuropsychologist. To assess global cognitive functioning, all participants were administered with the Mini-Mental State Examination (Folstein et al., 1975; Italian version Magni et al., 1996) and the Clock Drawing Test (Rouleau et al., 1992; Italian version Siciliano et al., 2016). Also, to the assess the global cognitive functioning focusing on the executive domain, the Frontal Assessment Battery (Dubois et al., 2000; Italian version Appollonio et al., 2005) was used.

Considering the impact of OSA syndrome on alertness, the cognitive abilities of attentional resources and cognitive inhibition (Vaessen et al., 2015) were measured through a computerized version of the Flanker’s Test (PEBL Psychological Test Battery) (Mueller and Piper, 2014). This computerized test measures the ability to suppress responses when inappropriate in a particular [context](https://en.wikipedia.org/wiki/Context_(language_use)), i.e. the response inhibition. Traditionally, to study the Flanker’s effect, participants are asked to detect stimuli (i.e. targets) presented centrally, while ignoring stimuli (i.e. the flankers) presented spatially close to them. However, flankers are processed by brain, even though participants are explicitly instructed to ignoring them. The flankers affect the behavioral responses to the central target (i.e. attentional capture) positively, if they are consistent with the target response, or negatively, if they are incompatible with the target (Kramer et al., 1999; Diedrichsen et al., 2000). In the PEBL Psychological Test Battery (Mueller, 2012; Mueller and Piper, 2014), the target is flanked by non-target stimuli which corresponded either to the same directional (right vs left) response as the target (congruent condition), to the opposite response (incongruent condition), or to neither (neutral condition). Also, there is the no-flanker condition, in which no flanker is shown. Overall, in the version used in this study, 160 trials were tested, 40 for each condition. Moreover, preliminary participants performed 8 run-in trials. Further technical details can be found online (http://pebl.sourceforge.net) and in Mueller and Piper (2014). For each trial, the reaction time in milliseconds (ms) and the level of accuracy (expressed in percentage) were detected (Scarpina et al., 2020). According to the Flanker’s effect, participants should respond faster and more accurate when target and flanker were congruent (i.e. both pointed towards the same, right or left, direction) compared with the incongruent condition (target and flanker pointed towards opposite directions), while response latencies in neutral condition and no flanker condition should be intermediate (Diedrichsen et al., 2000).

We followed the same procedure describe in Scarpina and colleagues (2020). From the collected data, we excluded the data relative to all those trials in which the RTs was out of the range of the two standard deviations from the RT mean in the corresponded experimental condition: overall, 14.85 % of trials were excluded. For each experimental condition (neutral, congruent, incongruent and single), we computed the *RTs* in milliseconds for the valid trials (i.e. in the case of correct answers) and the percentage of *Accuracy*.

**Subjective evaluation of sleeping**. Three questionnaires, which are routinely adopted in clinical settings for the self-description of sleep characteristics, were used in this study. We used the Italian version (Manni et al., 1999) of the Epworth Sleepiness Scale (Johns, 1991), that is a self-administered form with eight items, investigating tendency to fall asleep in daily life. Each item can score from zero to three points; the final score ranges from zero to 24, with higher scores indicating more daytime sleepiness. Scores over ten are generally representative of pathological daily sleepiness. We used also the Italian version (Curcio et al., 2013) of the self-rated questionnaire Pittsburgh Sleep Quality Index (Buysse et al., 2005) to describe the sleeping activity in terms of: quality, onset latency, duration, efficiency, sleep disturbances, use of sleeping medication, and daytime dysfunction. The global score is ranged between zero and 21; higher the score, higher the sleeping difficulties. Finally, we adopted the Italian version (Romigi et al., 2015) of Stanford Sleepiness Scale (Hoddes, 1975) which allows to quantify the subjective level of alertness and sleepiness throughout the day. For each hour of the day, participants rate their sensations from one (i.e. feeling active, vital, alert or wide awake) to seven (i.e. no longer fighting sleep, sleep onset soon, having dream-like thought). In Figure S1, we showed the data about our sample.

**Figure S1.** About the Stanford Sleepiness Scale, we report mean and standard deviation for each time hour; vertical lines represent standard deviation. a.m. = ante meridiem; p.m. = post meridiem.

**
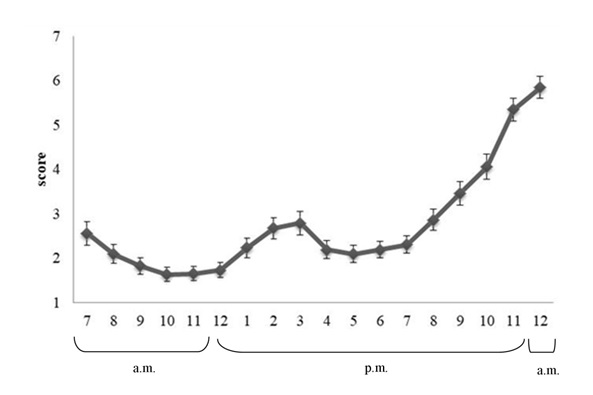
**

**Polysomnography.** We adopted the procedure that is routinely applied in our hospital (i.e. Zibetti et al., 2017), that is line with the international standard. All participants underwent a full-night polysomnography in the sleep laboratory in a quiet room. Participants were allowed to maintain their regular sleep habits and timing. During the examination, electrocardiogram activity, respiratory effort by thoracic and abdominal strain gauges, nasal air-flow by nasal cannula, snoring by a microphone, arterial oxyhemoglobin using a pulse oximeter with finger probe were assessed. Conventional sleep analyses, respiratory parameters, and arousal were performed independently by two clinicians experienced in sleep staging, following international guidelines (Iber et al., 2007). Specifically, the Apnoea–Hypopnoea Index (AHI), computed according to the number of [apnea](https://en.wikipedia.org/wiki/Apnea) and [hypopnea](https://en.wikipedia.org/wiki/Hypopnea) events per hour of sleep, were adopted as index of sleep apnea severity. Moreover, the following indexes were computed: the number of [hypopnea](https://en.wikipedia.org/wiki/Hypopnea) events per hour of sleep; the number of [apnea](https://en.wikipedia.org/wiki/Apnea) events per hour of sleep; the number of blood oxygen desaturation event per hour of sleep, the SpO2<90% (peripheral capillary oxygen saturation), the mean minimum oxygen saturation, and the minimum oxygen saturation (Berry et al., 2012).

**Procedure.** Participants were approached the day after their admission in our hospital by one member of the research group, if they satisfied the inclusion criteria. The experimenter illustrated the main goal of the study to the participants. After the informed consent signature, demographical information was collected. During the second night after the admission, the polysomnography was run. In the successive two days, participants filled out the questionnaires (duration: 1 hour) and performed the neuropsychological assessment (duration: 30 minutes).

**SUPPLEMENTAL ANALYSES AND RESULTS.**

**Comparisons between our sample and normative data.**

Any difference between our sample’s score and the normative data (i.e. when available in the seminal articles) for each psychological and neuropsychological tests was verified through an independent sample t-test.

**Temperament and Character Inventory.** The 69.23 % of our participants reported a higher score relative to the temperamental trait of persistence in comparison with the other traits; the 11.69 % of the participants, in the harm avoidance scale, as well as the 11.69 % in the novelty seeking scale, finally, only the 7.69 % of the participants, in the reward dependence scale. When we compared the scores relative to the Temperament and Character Inventory with the normative sample (N = 740; all individuals older than 18 years; *novelty seeking* mean score = 98.5; SD = 12.9; *harm avoidance* mean score = 96.4; SD = 14.4; *reward dependence* mean score = 101.4; SD = 13; *persistence* mean score = 116.3; SD = 14.4; *self-directedness* mean score = 139.1; SD = 16.5; *cooperativeness* mean score = 134.9; SD =13.9; *self-transcendence* mean score = 64.9; SD =14.5) (Martinotti, et al., 2008), a significant difference emerged in the scales relative to the *novelty seeking* temperament [t(790) = 1.82; p = 0.007; d’ = 0.25]. Instead, no difference was observed about the other temperamental traits [*harm avoidance* t(790) = 0.54; p = 0.58; d’ = 0.08; *reward dependence* t(790) = 0.06; p = 0.94; *d’* = 0.009; *persistence* t(790) = 1.56; p = 0.12; d’ = 0.23] and the three scales relative to the character [*self-directedness* t(790) = 1.59; p = 0.11; d’ = 0.24; *cooperativeness* t(790) = 0.1; p = 0.92; *d’* = 0.01; *self-transcendence* t(790) = 1.92; p = 0.059; d’ = 0.27].

**Neuropsychological tests.** We observed a significant difference [t(119) = 2.14; p = 0.03; d’ = 0.36] between our sample’s score and the normative data (n = 71; mean age = 65-69; mean score = 27.9; SD = 2) (Magni et al., 1996) in the Mini Mental State Examination. Our participants reported a higher score, meaning a better performance; however, it should be noted that our sample was younger in comparison with the normative sample.
No difference emerged when our sample was compared with the Italian normative data (n = 159; mean score = 8.56; SD = 1.79) (Siciliano et al., 2016) about the Clock Drawing Test [t(79) = 1.1; p = 0.27; d’ = 0.18] or with the Italian normative data (n = 66; mean score = 16.8; SD = 1.2) (Appollonio et al., 2005) relative to the Frontal Assessment Battery [t(99) = 0.49; p = 0.62; d’ = -0.09]. About the Flanker’s test, no normative data are available in literature. With the aim to verify if there the Flanker’s effect emerged in our sample’s performance, we performed a repeated measure Anova with the factor *Condition* (neutral, congruent, incongruent and single), independently for the RT and the percentage of *Accuracy*. When we considered the RT in milliseconds, we observed a main effect of *Condition* F(3, 147) = 172; p < 0.001; partial eta-squared p = 0.77]; specifically according to the post hoc estimated marginal means comparisons Bonferroni-corrected, the presence of an incongruent or congruent flanker affected the detection velocity in comparison with the conditions in which the flanker was not shown or it was neutral (Figure S2, left panel).

**Figure S2.** About the Flanker’s Test, we report mean and standard error (vertical lines) for each experimental condition (x-axis) for the Reaction Time (RT) expressed in milliseconds (left panel) and the Accuracy in percentage (right panel). * indicates a significant difference (p < 0.05), according to the post hoc analyses.


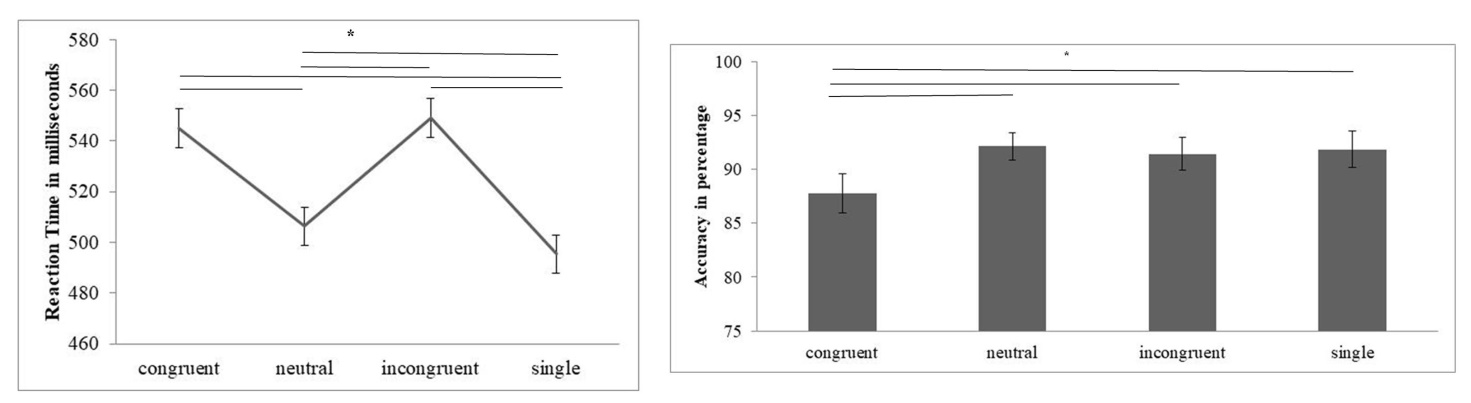


The main effect of *Condition* emerged also about the level of *Accuracy* [F(3.147) = 9.09; p < 0.001; partial eta squared = 0.15]; in this case, the presence of a congruent flanker decreased the level of accuracy in comparison with the other experimental conditions, in disagreement with the expected effect (Figure S2, right panel). Overall, these results confirmed the presence of attentional difficulties in our sample. For the main analyses, reported in the main article, for both the RT and the level of Accuracy, we computed an index, according to the following formula:

*Index = incongruent / mean (congruent, neutral and single).*

The index allowed us to describe the subjective level of sensitivity to the Flanker’s effect. About the RT, values higher than 1 suggested lower answer’s speed in the incongruent condition compared with the other conditions. About the level of Accuracy, values lower than 1 suggested lower level of accuracy in the incongruent condition compared with the other conditions. This index represented the subjective level of sensitivity to the Flanker’s effect. Means and standard deviations about the index relative to the RTs and the Accuracy were reported in Table 1 (main article). In conclusion, since we did not observed the expected cognitive performance in our sample, we might hypothesized that our participants suffered from attentional difficulties.

**Sleeping.** According to the polysomnography, 60% of our sample reported a severe sleep apnea syndrome, 20% a moderate level, 14% a mild level, and only 3% a normal level (Ruehland et al., 2009). About the Stanford Sleepiness Scale, we reported a significant difference [t(81) = 4.9; p < 0.001, d’ = 0.96] with the control sample reported by Romigi and colleagues (2015) (N = 80; mean score = 1.91, SD = 0.77): individuals reported higher scores in the time range between 1 p.m. and 4 p.m. (refer to the Supplemental Materials for details). About Pittsburgh Sleep Quality Index, the sample reported a significant higher score suggesting a worse sleep quality [t(52) = 7.21; p < 0.001; d’ = 1.65] in comparison with the data provided by Curcio and colleagues (2013) (N = 10; mean score 3.9; SD = 0.86). About the subjective sleep assessment, our sample reported a significantly higher score [t(75) = 4.96; p < 0.001; d’ = 0.97] in comparison with the normative data (N = 54; score = 4.4; SD = 2.8) (Manni et al., 1999) for the Epworth Sleepiness Scale.

**The relationship between OSA syndrome severity and investigated factors.**

We performed a correlational analyses between The Apnea–Hypopnea Index (AHI), that is a neurophysiological parameter relative to the severity of [sleep apnea](https://en.wikipedia.org/wiki/Sleep_apnea). and the scores relative to the other measurements used in this work. According to the results, reported in Table S1, no significant relationship emerged.

**Table S1**. The relationship between the AHI index and BMI, the scores relative to the personality (i.e. Temperament and Character Inventory), cognitive functioning (i.e. neuropsychological tests), and the subjective characteristics (self-report questionnaires) about sleeping.

|  | | Apnea–Hypopnea Index  (AHI) | |
| --- | --- | --- | --- |
| BMI | | ρ = 0.16 | p = 0.23 |
| **Personality**  Temperament and Character Inventory | | | |
| novelty seeking | | ρ = 0.1 | p = 0.46 |
| harm avoidance | | ρ = -0.12 | p = 0.39 |
| reward dependence | | ρ = -0.13 | p = 0.32 |
| persistence | | ρ = -0.09 | p = 0.51 |
| **Cognitive functioning** | | | |
| Mini Mental State Examination | | ρ = 0.15 | p = 0.27 |
| Clock Drawing Test | | ρ = 0.11 | p = 0.4 |
| Frontal Assessment Battery | | ρ = 0.08 | p = 0.5 |
| Flanker’s Test | RTs | ρ = -0.17 | p = 0.21 |
|  | Accuracy | ρ = -0.11 | p = 0.41 |
| **Subjective sleeping evaluation** | | | |
| Stanford Sleepiness Scale | | ρ = 0.1 | p = 0.46 |
| Pittsburgh Sleep Quality Index | | ρ = -0.07 | p = 0.61 |
| Epworth Sleepiness Scale | | ρ = 0.017 | p = 0.9 |

n = 52

**The relationship between BMI and investigated factors.**

We performed a correlational analyses between BMI, which represents a clinical index about the obesity severity, and the score relative to the all measurements used in this study. According to the results, reported in Table S2, no significant relationship emerged, except in the case of the novelty seeking temperament: as suggested by the negative ρ, a lower expression of novelty seeking temperament seemed to be significantly related to higher levels of obesity severity, (i.e., a higher BMI).

**Table S2**. The relationship between the BMI and the scores relative to the personality (i.e. Temperament and Character Inventory), cognitive functioning (i.e. neuropsychological tests), and the subjective characteristics (self-report questionnaires) about sleeping. We report again the relationship with the AHI index, as shown in Table S1.

|  | | BMI | |
| --- | --- | --- | --- |
| AHI index | | ρ = 0.16 | p = 0.23 |
| **Personality**  Temperament and Character Inventory | | | |
| novelty seeking | | ρ = -0.29 | **p = 0.03** |
| harm avoidance | | ρ = 0.01 | p = 0.91 |
| reward dependence | | ρ = 0.12 | p = 0.37 |
| persistence | | ρ = 0.18 | p = 0.19 |
| **Cognitive functioning** | | | |
| Mini Mental State Examination | | ρ = -0.01 | p = 0.92 |
| Clock Drawing Test | | ρ = -0.02 | p = 0.89 |
| Frontal Assessment Battery | | ρ = -0.23 | p = 0.1 |
| Flanker’s Test | RTs | ρ = 0.16 | p = 0.24 |
|  | Accuracy | ρ = -0.11 | p = 0.41 |
| **Subjective sleeping evaluation** | | | |
| Stanford Sleepiness Scale | | ρ = 0.14 | p = 0.3 |
| Pittsburgh Sleep Quality Index | | ρ = 0.03 | p = 0.83 |
| Epworth Sleepiness Scale | | ρ = 0.26 | p = 0.06 |

n = 52. In bold, significant relationships.

**Analysis relative to the difference in investigated factors between the different levels of sleep apnea severity.**

As reported in the main text, a value of AHI below of the cut-off of 5 was considered in the range of normality; values between the range 5≤AHI<15 defined a mild sleep apnea; the range 15≤AHI<30, a moderate apnea; values of AHI ≥ 30 defined a severe apnea (Ruehland et al., 2009). We observed that the majority of our participants (60 %) showed a severe sleep apnea; 20 % of participants, a moderate OSAS; 10 % of participants, a mild OSAS; and, 6 % of participants, no sleep apnea. Indeed, it should be considered that participants were recruited because of their referred symptoms, instead of the evidence of OSA syndrome at the polysomnography. We studied the difference at the global score relative to psychological well-being, personality, cognitive functioning, subjective sleep quality, and BMI, between the four level of sleep apnea (normal, mild, moderate, and severe) through an one-way between-subjects ANOVA. According to the results reported in Table S3, no difference emerged between the different level of sleep apnea in the investigated factors.

**Table S3.** Mean and standard deviation (SD) for each assessed factors are reported for the different levels of sleep apnea, along with the statistical results.

|  | | **Level of sleep apnea** | | | |  |
| --- | --- | --- | --- | --- | --- | --- |
|  | | **normal** | **mild** | **moderate** | **severe** |  |
| BMI | | 39.04 (33.81) | 42.19 (6.52) | 44.52 (8.54) | 46.52 (5.38) | F(3,49)=0.88; p = 0.45 |
| **Psychological well being:** Psychological General Well Being Index | | | | | | |
| anxiety | | 11.66 (8.62) | 11.28 (4.34) | 14.5 (3.68) | 14.73 (5.25) | F(3,49) = 1.11; p = 0.35 |
| depression | | 10.33 (5.03) | 9.14 (3.71) | 12.4 (2.27) | 11.33 (2.97) | F(3,49) = 1.64; p = 0.19 |
| positive well-being | | 8 (4) | 7 (3.78) | 10 (3.33) | 8.96 (4.13) | F(3,49) = 0.85; p = 0.47 |
| self-control | | 11 (2.64) | 8.57 (3.69) | 10.1 (3.28) | 9.96 (3.27) | F(3,49) = 0.5; p = 0.68 |
| general healthy | | 6.66 (3.51) | 7.42 (2.87) | 7.8 (3.52) | 7.76 (2.93) | F(3,49) = 0.13; p = 0.93 |
| vitality | | 6.33 (4.61) | 7.28 (5.49) | 9.8 (2.57) | 9.86 (4.42) | F(3,49) = 0.78; p = 0.5 |
| total score | | 54 (27.62) | 50.71 (21.53) | 64.6 (14.4) | 61.63 (19.61) | F(3,49) = 0.88; p = 0.45 |
| **Personality:** Temperament and Character Inventory | | | | | | |
| novelty seeking | | 74 (23,38) | 54 (32.37) | 57.3 (29.07) | 54.43 (28.04) | F(3,49) = 0.44; p = 0.72 |
| harm avoidance | | 85.66 (22.89) | 58.57 (30.59) | 53.5 (25.12) | 50.06 (21.26) | F(3,49) = 2.18; p = 0.1 |
| reward dependence | | 85.66 (30.82) | 69.57 (19.45) | 67.8 (22) | 64.4 (21.46) | F(3,49) = 0.91; p = 0.44 |
| persistence | | 114.66 (26.63) | 100.71 (24.89) | 105.6 (30.25) | 103.63 (17.98) | F(3,49) = 0.29; p = 0.82 |
| self-directedness | | 102 (53.45) | 69.14 (43.57) | 73.3 (50.78) | 64.83 (41.12) | F(3,49) = 0.68; p = 0.56 |
| cooperativeness | | 101.33 (31.65) | 86.42 (37.64) | 85.4 (37.28) | 77.96 (29.05) | F(3,49) = 0.6; p = 0.61 |
| self-transcendence | | 82.66 (4.5) | 69.42 (16.14) | 73.4 (13.21) | 68.23 (15.05) | F(3,49) = 1.08; p = 0.36 |
| **Cognitive functioning** | | | | | | |
| Mini Mental State Examination | | 27.66 (2.3) | 28 (2) | 28.88 (0.92) | 28.66 (1.15) | F(3,48) = 1.1; p = 0.35 |
| Clock Drawing Test | | 7.16 (3.01) | 8.78 (1.55) | 9.22 (1.27) | 8.95 (2.19) | F(3,48) = 0.81; p = 0.49 |
| Frontal Assessment Battery | | 15.33 (3.05) | 17.14 (0.69) | 16.44 (1.81) | 16.76 (1.22) | F(3,48) = 1.26; p = 0.29 |
| Flanker’s Test | RTs | 1.1 (0.02) | 1.07 (0.03) | 1.06 (0.03) | 1.05 (0.03) | F(3,47) = 1.88; p = 0.14 |
|  | Accuracy | 1.06 (0.02) | 0.99 (0.03) | 1.01 (0.01) | 1 (0.04) | F(3,47) = 1.97; p = 0.13 |
| **Subjective sleeping evaluation** | | | | | | |
| Stanford Sleepiness Scale | | 3.2 (0.84) | 3.33 (1.46) | 2.31 (0.72) | 2.76 (0.95) | F(3,47) = 1.62; p = 0.19 |
| Pittsburgh Sleep Quality Index | | 4 (1) | 5.28 (3.77) | 3.9 (2.37) | 4.13 (2.35) | F(3,47) = 0.47; p = 0.7 |
| Epworth Sleepiness Scale | | 13.66 (4.5) | 8.42 (3.95) | 6 (4.18) | 8.73 (5.8) | F(3,47) = 0.47 p = 0.7 |

**Additional References in Supplemental Materials.**

Berry, R.B., Budhiraja, R., Gottlieb, D.J., et al. (2012). Rules for scoring respiratory events in sleep: update of the 2007 AASM Manual for the Scoring of Sleep and Associated Events. Deliberations of the Sleep Apnea Definitions Task Force of the American Academy of Sleep Medicine. *J. Clin. Sleep Med.* 8(5), 597-619. doi:10.5664/jcsm.2172

Diedrichsen J, Ivry RB, Cohen A, Danziger S. (2000). Asymmetries in a unilateral flanker task depend on the direction of the response: the role of attentional shift and perceptual grouping*. J. Exp. Psychol. Hum. Percept. Perf.* 26(1): 113–126. doi:10.1037//0096-1523.26.1.113

Iber, C., Ancoli-Israel, S., Chesson, A. &, Quan, S.F. for the American Academy of Sleep Medicine (2007). *The AASM manual for the scoring of sleep and associated events: rules, terminology and technical specifications*. Westchester: American Academy of Sleep Medicine.

Kramer, A.F., Hahn, S., Irwin, D.E., Theeuwes, J. (1999). Attentional capture and aging: implications for visual search performance and oculomotor control. *Psychol. Aging,* 14(1): 135–154. doi:10.1037//0882-7974.14.1.135

Mueller, S.T. (2012). *The PEBL Manual*, Version 0.13. Lulu Press. 2012. ISBN 978-0557658176.

Scarpina F, D’Agata F, Priano L, & Mauro A. (2020). Difference between young and old adults’ performance on the Psychology Experiment Building Language (PEBL) Test Battery: what is the role of familiarity with technology in cognitive performance? *Assessment* (in press) doi: 10.1177/1073191120918010.
